# The Role of Systemic Arterial Stiffness in Open-Angle Glaucoma with Diabetes Mellitus

**DOI:** 10.1155/2015/425835

**Published:** 2015-10-18

**Authors:** Seong Hee Shim, Chan Yun Kim, Joon Mo Kim, Da Yeong Kim, Yang Jae Kim, Jeong Hun Bae, Ki Chul Sung

**Affiliations:** ^1^Department of Ophthalmology, Kangbuk Samsung Hospital, Sungkyunkwan University School of Medicine, 29 Saemunan-ro, Jongno-gu, Seoul 110-746, Republic of Korea; ^2^Department of Ophthalmology, Yonsei University College of Medicine, Seoul, Republic of Korea; ^3^Division of Cardiology, Department of Medicine, Kangbuk Samsung Hospital, Sungkyunkwan University School of Medicine, 29 Saemunan-ro, Jongno-gu, Seoul 110-746, Republic of Korea

## Abstract

*Purpose.* To investigate the role of systemic arterial stiffness in glaucoma patients with diabetes mellitus (DM).* Design.* Retrospective, cross-sectional study.* Participants.* DM subjects who underwent brachial-ankle pulse wave velocity (baPWV) were recruited.* Methods.* Glaucoma patients (*n* = 75) and age-matched control subjects (*n* = 92) were enrolled. Systemic examination including BaPWV and detailed eye examination were performed. The glaucoma group was divided into subgroups of normal tension glaucoma (NTG, *n* = 55) and primary open-angle glaucoma (POAG) based on an IOP of 21 mmHg. BaPWV was used to stratify the population into 4 groups based on the rate. Stepwise multiple logistic regression analysis by baPWV quartiles was used to compare the glaucoma group with the control group.* Main Outcome Measures. *BaPWV in glaucoma with DM patients.* Results.* Faster baPWV was positively associated with glaucoma (odds ratio: 3.74; 95% CI: 1.03–13.56, stepwise multiple logistic regression analysis) in patients with DM. Increasing baPWV was also positively associated with glaucoma (*p* for trend = 0.036). The NTG subgroup showed similar results to those of the glaucoma group.* Conclusions.* In this study, increased arterial stiffness was shown to be associated with glaucoma and may contribute to the pathogenesis of glaucoma in DM patients.

## 1. Introduction

Glaucoma is defined as chronic progressive optic nerve damage and regarded as a multifactorial disease caused by genetic, local, systemic, and environmental factors [1]. The traditional theoretical mechanisms of the development of glaucoma include the mechanical theory, which emphasizes the role of high intraocular pressure (IOP) [2], and the ischemic theory, which focuses on disturbance of blood flow [3]. Recently, many studies have demonstrated systemic risk factors for glaucoma, like metabolic syndrome [4], helicobacter pylori [5], diabetes mellitus (DM) [6], and thyroid disease [7].

The Beaver Dam eye study [6], national health care study [8], and meta-analysis study [9, 10] have suggested that DM is significantly associated with increased IOP and glaucoma. Thus, this is an area of ongoing investigation and debate. Another possibility is that glaucoma may be associated not just with DM but also with its secondary effect on systemic or local factors. The hypothetical mechanisms of DM as a risk factor for glaucoma include the alteration of biochemical pathways, increased oxidative stress by lipid metabolism, and blood flow alteration by the disturbance of autoregulation of vascular endothelium [11].

Some studies about migraine [12], peripheral blood flow [13], or systemic blood flow [14] have suggested that the disturbance of systemic circulation is a possible risk factor for glaucoma. Increased arterial stiffness, which can occur as a consequence of arteriosclerosis, is associated with DM and systemic hypertension, which may in part explain the increased cardiovascular disease risk observed in these conditions. However, there are few studies on the association between glaucoma and arterial stiffness and its potential impact on retinal circulation. Furthermore, the relationship between systemic circulation abnormalities such as DM, systemic hypertension, and glaucoma remains unclear.

In this study, we evaluated the role of systemic arterial stiffness in DM patients with glaucoma.

## 2. Materials and Methods

This study was a retrospective chart review. Subjects were recruited from the glaucoma outpatient clinic at Kangbuk Samsung Hospital, Seoul, Korea, from January to December 2013. All subjects were new patients who were referred from or subjects who were followed in the DM clinic of the same hospital. This study was performed in accordance with the Declaration of Helsinki and was approved by the Institutional Review Board and ethics committee of Kangbuk Samsung Hospital in Seoul, Korea.

For inclusion in this study, all participants met the following criteria: the logarithm of the minimal angle of resolution best corrected visual acuity (logMAR BCVA) was 0.70 or better, a spherical equivalent within 6 diopters (D) and a cylinder within 3 D, the presence of an open-angle on gonioscopic examination, and reliable VF test results. Glaucoma was diagnosed by a glaucoma specialist using the following criteria: presence of typical glaucomatous optic disc neuropathy including rim thinning or notching in the inferior or superior temporal area of the optic nerve head, corresponding glaucomatous VF loss including paracentral, arcuate scotomas, or a nasal step [15], and no apparent secondary cause of glaucomatous optic neuropathy. Determination of the diurnal IOP consisted of measuring the IOP every 150 minutes from 9 a.m. to 5 p.m. at the first visit in the absence of glaucoma medications. Based on this diurnal IOP profile, we divided the glaucoma group into normal tension glaucoma (NTG, less than 21 mmHg peak IOP) and primary open-angle glaucoma (POAG).

Exclusion criteria were active ocular disease, proliferative diabetic retinopathy (PDR) in any eye, use of other ocular medications or therapies that might have a substantial effect on IOP, and history of ocular surgery. After detailed ophthalmic evaluation including fundus exam after pupil dilation, the right eye was chosen for inclusion in cases in which both eyes of the patient were eligible for the study. For the control group (patients without glaucoma), age-matched subjects were selected.

All subjects underwent a detailed ophthalmic examination including medical and ophthalmic histories, best corrected visual acuity (BCVA) testing, IOP measurements by Goldmann applanation tonometry, VF testing (Zeiss-Humphrey, San Leandro, CA), color disc photography (Visucam Pro NM model; Carl Zeiss Meditec, Inc.), and optical coherence tomography (Cirrus OCT, Carl Zeiss Meditec, Inc., Dublin, CA). Systemic examinations were performed including measurements of brachial-ankle pulse wave velocity (baPWV) (automatic waveform analyzer: VP-1000, Colin Co., Komaki, Japan) for assessment of systemic arterial stiffness. This instrument records baPWV (cm/sec), electrocardiogram, systemic blood pressure (BP), pulse pressure (PP), and ankle-brachial index (ABI). The baPWV was measured after the subject was allowed to rest for at least 5 minutes. Carotid intima-media thickness (IMT) was also measured at the common carotid artery, bulb, and internal carotid artery with carotid sonography (Vivid E9, GE Healthcare, WI, USA). Body mass index (BMI) was calculated as weight in kilograms divided by the height in meters squared. Laboratory blood tests including total cholesterol, triglyceride (TG), and fasting blood glucose (FBS) were performed. And hemoglobin A1c (HbA1C) were performed as index which represent severity of DM.

### 2.1. Statistical Methods

Baseline demographic information and clinical parameters were compared between groups using independent sample *t*-tests or one-way analysis of variance (ANOVA) with the Bonferroni post hoc test for continuous variables and Chi-square tests or Fisher's exact tests for categorical variables.

Odds ratios (OR) with 95% confidence intervals (95% CI) were estimated using logistic regression models. BaPWV was stratified into 4 groups based on the rate. Following univariate analyses, multivariate logistic regression analyses were performed to identify independent risk factors for glaucoma using backward selection based on the likelihood ratio.

All data were analyzed using SPSS statistical software system version 21.0 (IBM SPSS, Inc., Chicago, Illinois, USA). A *p* value < 0.05 was considered to be statistically significant.

## 3. Results

A total of 167 patients were enrolled in the study, 108 of whom were men and 59 of whom were women. The mean age was 59.26 ± 9.86 years (range, 34–79 years). Sixty-nine patients (41.3%) had hypertension.

Table 1 shows the demographics and clinical data of subjects. All subjects were divided into the glaucoma group (*n* = 75) or the control group (*n* = 92). There was no significant difference in demographics and clinical characteristics between the glaucoma and control groups. Mean baPWV of the glaucoma group was about 88.3 cm/sec (5.7%) faster than that of the control group (1638.73 ± 276.04 versus 1550.43 ± 256.39, independent sample *t*-tests, *p* = 0.035) Right baPWV of the glaucoma group was about 83.9 cm/sec (5.4%) faster than that of the control group (1633.16 ± 278.05 versus 1549.24 ± 260.54, independent sample *t*-tests, *p* = 0.048). Left baPWV of the glaucoma group was about 92.7 cm/sec (6.0%) faster than that of the control group (1644.31 ± 280.46 versus 1551.63 ± 258.36, independent sample *t*-tests, *p* = 0.029). There were not any significant differences in mean IOP, systolic and diastolic BP, PP, mean arterial pressure, ABI, IMT, BMI, total cholesterol, TG or FBS, and HbA1C between the glaucoma and control groups. Six patients (8.0%) have mild nonproliferative diabetic retinopathy (NPDR) in OAG group and 5 patients (5.4%) in control group. And moderate NPDR showed in 4 patients (5.3%) in OAG and 2 patients (2.2%) in control group. Severe NPDR showed in 2 patients (2.7% in OAG, 2.2% control group) in each group. There was no statistically significant difference of severity of diabetic retinopathy between the glaucoma and control groups.

Comparing of demographic and clinical data in NTG, POAG and control group showed in Table 2. There was no significant difference in systolic and diastolic BP, PP, mean arterial pressure, ABI, IMT, BMI, total cholesterol, TG or FBS, and HbA1C between the NTG and POAG groups except mean IOP. In comparison of baPWV among the three groups, mean baPWV of the NTG group was about 112.8 cm/sec (7.3%) faster than that of the control group (1663.25 ± 287.35 versus 1550.43 ± 256.39, independent sample *t*-tests, *p* = 0.02). However, mean baPWV of the POAG group was about only 20.9 cm/sec (1.3%) faster than that of the control group (1571.33 ± 235.76 versus 1550.43 ± 256.39, independent sample *t*-tests, *p* = 0.04).

Stepwise multiple logistic regression analysis was adjusted for age, gender, mean arterial BP, BMI, TG, HbA1C, and severity of diabetic retinopathy; it showed that a faster than 1740.5 cm/sec (fourth quartile) (OR adjusted for age, gender, mean arterial BP, BMI, TG, HbA1C, and severity of diabetic retinopathy: 3.74; 95% CI: 1.03–13.56, Table 3) was independently associated with glaucoma. Furthermore, increasing baPWV was an independent risk factor of glaucoma (*p* for trend = 0.036).

In subgroup analyses, the NTG group comprised 55 patients. The results were similar to the total analysis (Table 4). Stepwise multiple logistic regression model, the fourth quartile (baPWV above 1718 cm/sec) (OR adjusted for age, gender, mean arterial BP, BMI, TG, HbA1C, and severity of diabetic retinopathy: 4.26; 95% CI: 1.08–16.88) were independently associated with NTG (Table 4). Furthermore, increasing baPWV was an independent risk factor of NTG (*p* for trend = 0.025). There was no independently associated factor with POAG (Table 5).

## 4. Discussion

Our study showed that high baPWV was an independent risk factor for glaucoma in DM patients. Moreover, faster baPWV was positively associated with increasing OR of glaucoma. These findings suggest that arterial stiffness may be an independent risk factor for glaucoma. baPWV is a good surrogate measure of the effect of systemic arterial stiffness on ocular blood vessels, which may estimate ocular blood flow. In subgroup analysis, the NTG group showed similar results. Mrocxkowska et al. suggested that NTG and POAG patients showed similarly increased nocturnal systemic blood pressure variability (*p* = 0.01), peripheral arterial stiffness (*p* = 0.02), carotid intima-media thickness (*p* = 0.04), and reduced ocular perfusion pressure (*p* < 0.001). Their study showed that POAG and NTG patients exhibit similar alterations in ocular and systemic circulation in the early stages of their disease process [16]. Arterial stiffness may lead to the disruption of ocular autoregulation [17]. In this study, there was no significantly different clinical data including baPWV between the NTG and POAG groups except mean IOP. Mean baPWV of the NTG group was about 7.3% faster than that of the control group. However, mean baPWV of the POAG group was about only 1.3% faster than that of the control group. These results suggested that arterial stiffness is more associated with NTG than with POAG.

PWV was known as a good marker of systemic arterial stiffness as it reflects local as well as systemic circulation [18, 19]. Furthermore, the baPWV measurement unit was technically simple and could be used to check the status of both the central arteries and peripheral arteries. A previous study found that baPWV had good validity and reproducibility for assessing vascular damage [20]. Although baPWV was a good marker of systemic arterial stiffness, the results can still be affected by age [17], gender [17], BP [21], BMI [17], and plasma triglycerides [17]. To rule out the effect of BP variation, baPWV was checked routinely in the resting state after at least 10 minutes of break in all subjects. In addition, we adjusted our results for age, gender, BP, BMI, and plasma triglycerides to exclude confounding factors. In our study, there were no significant associations between HTN, SBP, DBP, PP, ABI, IMT, BMI, total cholesterol level, triglyceride level, FBS, HbA1C, severity of diabetic retinopathy, and glaucoma. The severity of DM complications of our subjects was no more than severe NPDR, which suggests that lack of evident systemic complications may be an important factor. Severity of diabetic retinopathy was not different between glaucoma and control group. Furthermore, HbA1C did not show statistically significant difference between the two groups. After adjusting for severity of diabetic retinopathy and HbA1C, baPWV was independent risk factor of glaucoma. These results would support the concept of arterial stiffness associated with glaucoma independently from severity of diabetic retinopathy or severity of DM.

Glaucoma may be associated with an alteration in factors related to ocular blood flow and a breakdown of autoregulation [22]. In glaucoma patients with impaired vascular autoregulation of ocular vessels, PP beyond critical range can be hazardous to the ocular blood vessels and their autoregulation [23–25]. In cases of impaired ocular vascular autoregulation, ischemia and optic nerve damage can develop due to low perfusion pressure caused by rising IOP and low diastolic BP [26]. Grunwald et al. reported the presence of decreased optic nerve blood flow in glaucoma patients. In their study, patients with more advanced glaucomatous damage in the visual field and cup-to-disc ratio tended to exhibit worse optic nerve blood flow, suggesting that decreased optic nerve blood flow might be associated with progression of functional and morphologic glaucomatous damage [23]. Michelson et al. suggested that POAG patients showed significantly decreased optic nerve head blood flow and juxtapapillary blood flow compared with an age-matched control group [27]. However, this decreased juxtapapillary blood flow was not significantly different between glaucoma patients with and without treatment [27]. Schwartz found that the circulatory defects in the optic disk and retina were present in glaucoma and ocular hypertension subjects and that these findings were correlated with glaucoma progression [28]. Several studies have shown an association between the disturbance of systemic circulation and glaucoma [12–14].

Some studies have suggested the relationship between systemic arteriosclerosis and retinal microvascular abnormalities. For example, hypertension has been associated with retinal microvascular abnormalities including retinopathy, focal arteriolar narrowing, and arteriovenous nicking [29]. After adjusting for age, gender, race, mean arterial blood pressure, and antihypertensive medication use, retinopathy was associated with prevalent coronary heart disease (OR, 1.7), prevalent myocardial infarction (OR, 1.7), prevalent stroke (OR, 2.0), presence of carotid artery plaque (OR, 1.9), increased intima-media thickness of the common carotid (OR, 2.3; fourth versus first quartile), and internal carotid (OR, 1.8; fourth versus first quartile) arteries [29]. Wong et al. reported an association between retinal arteriolar narrowing and risk of coronary heart disease [30]. Another study suggested that a wider retinal venule was associated with an increased relative risk of coronary heart disease-related death [31]. In that study, a smaller arteriole to venule ratio and narrower arterioles were significantly associated with coronary heart disease-related death. Together, the association of these retinal microvascular abnormalities to systemic arteriosclerosis emphasizes that systemic arteriosclerosis can alter the microvascular environment of the optic nerve head.

Relation between the systemic vascular function and glaucoma also has been raised. Several studies reported association of endothelial dysfunction and glaucoma. Su et al. reported impaired endothelium-dependent flow-mediated vasodilation in patients with NTG [32]. Another study showed glaucoma patients have impaired endothelium-dependent flow-mediated vasodilation and NTG patients have lower endothelium-dependent flow-mediated vasodilation than those with POAG [33]. These results would support the concept of a systemic vascular dysfunction in OAG patients, especially with NTG. And these results correspond with this study results that faster baPWV in glaucoma (especially in NTG) than control group.

The causes of the increased systemic arterial stiffness may be because of the advanced glycation endproducts (AGEs) and nitric oxide (NO) dysregulation in DM. Glucose interacts with the free amino acid residues of proteins [34]. This initial reaction leads to the formation of intermediate reversible Amadori products. The Amadori products change to the irreversible AGEs ultimately. The AGE can deposit on collagen the arterial wall and cause pathologic cross-linking. AGE-mediated cross-links have a high resistance to enzymatic proteolysis and a low degradation rate, which may contribute to an increased collagen content in arterial walls, characteristic of aging, and accelerated in DM [35]. NO has vasodilatory, antioxidant, antiplatelet, and anti-inflammatory properties [36]. In the insulin-resistant state, NO synthase activation is impaired and it may contribute to endothelial dysfunction through interfering with NO-mediated vasodilatation [37].

Increased systemic arterial stiffness may cause decreased optic nerve blood flow and alter the vascular environment of the optic nerve, making it vulnerable to insult. However, there are few studies on the association between systemic arteriosclerosis and retinal circulation in patient with glaucoma. In the Rotterdam Study, participants with an increased PWV, indicative of high arterial stiffness, had a higher prevalence of POAG, but the results were not statistically significant (the ORs were adjusted for age, sex, DM, cholesterol level, BMI, smoking, mean arterial BP, and use of systemic antihypertensive medication; both definite and probable glaucoma cases were included in the case group) [26]. The progression of arteriosclerosis may result in blood vessels with small lumens, increased resistance of peripheral vessels, endothelial dysfunction, and decreased oxygenation. It may cause an alteration in circulatory hemodynamics. Since quantitative analysis of ocular arteriolosclerosis is difficult, the relationship between ocular arteriolosclerosis and local/systemic circulation has not been clearly defined.

In conclusion, DM patients with glaucoma showed a higher brachial-ankle pulse wave velocity, which is generally recognized as a marker of systemic arterial stiffness. Our results suggest that systemic arterial stiffness may contribute to the pathogenesis of glaucoma in DM patients. Thus, a portion of DM patients who had high-grade systemic arterial stiffness may be vulnerable to glaucoma. This is the first study of the role of systemic arterial stiffness in glaucoma in DM patients. However, this disruption of vascular autoregulation may be correlated with various factors. Further studies are needed to better understand the microcirculation which affects the optic nerve head.

## Figures and Tables

**Table 1:** Comparison of demographic and clinical data.

	OAG (*n* = 75)	Control (*n* = 92)	*p* value
Gender (male : female)	47 : 28	61 : 31	0.625
Mean age (years)	59.03 ± 10.15	59.46 ± 9.68	0.78
Hypertension	29 (38.7%)	40 (43.5%)	0.53
Mean IOP (mmHg)	14.55 ± 4.08	13.93 ± 2.85	0.26
Systolic blood pressure (mmHg)	126.80 ± 18.10	125.28 ± 13.77	0.54
Diastolic blood pressure (mmHg)	75.87 ± 9.98	74.87 ± 9.53	0.51
Pulse pressure (mmHg)	50.93 ± 12.63	50.41 ± 9.38	0.76
Mean arterial pressure (mmHg)	93.56 ± 11.83	91.67 ± 10.21	0.30
Mean baPWV (cm/s)	1638.73 ± 276.04	1550.43 ± 256.39	0.035
baPWV, Rt. (cm/s)	1633.16 ± 278.05	1549.24 ± 260.54	0.048
baPWV, Lt. (cm/s)	1644.31 ± 280.46	1551.63 ± 258.36	0.029
ABI, Rt.	1.14 ± 0.08	1.12 ± 0.11	0.26
ABI, Lt.	1.14 ± 0.09	1.12 ± 0.10	0.21
Intima-media thickness (IMT)			
CCA, Rt. (mm)	0.71 ± 0.13	0.68 ± 0.14	0.28
CCA, Lt. (mm)	0.71 ± 0.16	0.73 ± 0.15	0.53
Bulb, Rt. (mm)	0.86 ± 0.23	0.83 ± 0.17	0.43
Bulb, Lt. (mm)	0.80 ± 0.18	0.86 ± 0.23	0.21
ICA, Rt. (mm)	0.56 ± 0.13	0.55 ± 0.08	0.58
ICA, Lt. (mm)	0.54 ± 0.07	0.57 ± 0.08	0.20
Body mass index (BMI)	25.80 ± 6.08	25.19 ± 3.46	0.44
Total cholesterol	169.04 ± 41.72	161.08 ± 39.14	0.21
Triglyceride	179.37 ± 2266.87	141.02 ± 167.58	0.28
Fast blood glucose	145.47 ± 55.84	133.59 ± 43.33	0.12
HbA1C (%)	6.97 ± 1.23	7.00 ± 1.32	0.87
Diabetic retinopathy			0.639
No diabetic retinopathy	63 (84.0%)	83 (90.2%)	
Mild NPDR	6 (8.0%)	5 (5.4%)	
Moderate NPDR	4 (5.3%)	2 (2.2%)	
Severe NPDR	2 (2.7%)	2 (2.2%)	

Data are expressed as the mean ± standard deviation (SD) or frequency (%).

baPWV: brachial-ankle pulse wave velocity; ABI: ankle-brachial index; CCA: common carotid artery; ICA: internal carotid artery; NPDR: nonproliferative diabetic retinopathy.

**Table 2:** Comparison of demographic and clinical data in NTG, POAG, and control group.

	NTG (*n* = 55)	POAG (*n* = 20)	Control (*n* = 92)	*p* value
Gender (male : female)	35 : 20	12 : 8	61 : 31	0.85
Mean age (years)	60.02 ± 56.30	56.30 ± 10.91	59.46 ± 9.68	0.34
Hypertension	22 (40.0%)	7 (35.0%)	40 (43.5%)	0.76
Mean IOP (mmHg)	13.15 ± 2.64	18.38 ± 4.90	13.93 ± 2.85	<0.001
Systolic blood pressure (mmHg)	126.91 ± 18.50	126.50 ± 17.41	125.28 ± 13.77	0.83
Diastolic blood pressure (mmHg)	75.09 ± 9.56	78.00 ± 11.04	74.87 ± 9.53	0.42
Pulse pressure (mmHg)	51.82 ± 12.95	48.50 ± 11.68	50.41 ± 9.38	0.49
Mean arterial pressure (mmHg)	92.36 ± 11.73	99.03 ± 11.16	91.67 ± 10.21	0.09
Mean baPWV (cm/s)^*∗*^	1663.25 ± 287.35	1571.33 ± 235.76	1550.43 ± 256.39	0.04
baPWV, Rt. (cm/s)	1659.27 ± 291.81	1561.35 ± 227.36	1549.24 ± 260.54	0.052
baPWV, Lt. (cm/s)^*∗*^	1667.22 ± 288.66	1581.30 ± 252.65	1551.63 ± 258.36	0.042
ABI, Rt.	1.13 ± 0.08	1.16 ± 0.07	1.12 ± 0.11	0.23
ABI, Lt.	1.13 ± 0.09	1.16 ± 0.08	1.12 ± 0.10	0.17
Intima-media thickness (IMT)				
CCA, Rt. (mm)	0.72 ± 0.13	0.65 ± 0.08	0.68 ± 0.14	0.27
CCA, Lt. (mm)	0.72 ± 0.17	0.65 ± 0.11	0.73 ± 0.15	0.49
Bulb, Rt. (mm)	0.88 ± 0.25	0.74 ± 0.06	0.83 ± 0.17	0.18
Bulb, Lt. (mm)	0.81 ± 0.19	0.74 ± 0.11	0.86 ± 0.23	0.37
ICA, Rt. (mm)	0.56 ± 0.13	0.57 ± 0.10	0.55 ± 0.08	0.84
ICA, Lt. (mm)	0.55 ± 0.07	0.52 ± 0.06	0.57 ± 0.08	0.33
Body mass index (BMI)	26.09 ± 6.90	25.02 ± 2.81	25.19 ± 3.46	0.50
Total cholesterol	164.80 ± 41.23	180.70 ± 41.88	161.08 ± 39.14	0.14
Triglyceride	174.93 ± 287.37	191.60 ± 206.09	141.02 ± 167.58	0.51
Fast blood glucose	148.02 ± 61.13	138.45 ± 38.10	133.59 ± 43.33	0.23
HbA1C (%)	7.08 ± 1.33	6.67 ± 0.86	7.00 ± 1.32	0.47
Diabetic retinopathy				0.39
No diabetic retinopathy	46 (83.6%)	17 (85.0%)	83 (90.2%)	
Mild NPDR	3 (5.5%)	3 (15.0%)	5 (5.4%)	
Moderate NPDR	4 (7.3%)	0 (0%)	2 (2.2%)	
Severe NPDR	2 (3.6%)	0 (0%)	2 (2.2%)	

Data are expressed as the mean ± standard deviation (SD) or frequency (%).

baPWV: brachial-ankle pulse wave velocity; ABI: ankle-brachial index; CCA: common carotid artery; ICA: internal carotid artery; NPDR: nonproliferative diabetic retinopathy. ^*∗*^ *p* < 0.05 between NTG and control.

**Table 3:** Multivariate logistic regression analysis by quartile baPWV.

Risk factor	Odds ratio	95% confidence interval		*p* value
Age	0.98	0.94	1.02	0.30
Gender				
Female	1			
Male	1.03	0.48	2.20	0.94
Mean arterial pressure	0.99	0.96	1.03	0.62
Body mass index	1.03	0.96	1.12	0.40
Triglyceride	1.00	0.999	1.002	0.36
HbA1C	1.00	0.77	1.31	0.98
Diabetic retinopathy				
No diabetic retinopathy (*n* = 63)	1			
Mild NPDR (*n* = 6)	1.32	0.35	4.97	0.69
Moderate NPDR (*n* = 4)	2.55	0.43	15.24	0.31
Severe NPDR (*n* = 2)	1.28	0.17	9.88	0.81
baPWV (cm/s)^*∗*^				
baPWV ≤ 1390 (*n* = 14)	1			
1390 < baPWV ≤ 1549.5 (*n* = 17)	1.79	0.64	5.05	0.27
1549.5 < baPWV ≤ 1740.5 (*n* = 21)	3.07	0.98	9.63	0.054
1740.5 < baPWV (*n* = 23)	3.74	1.03	13.56	0.045

^*∗*^ *p* for trend = 0.036.

**Table 4:** Multivariate logistic regression analysis by quartile baPWV in NTG.

Risk factor	Odds ratio	95% confidence interval		*p* value
Age	0.98	0.94	1.03	0.36
Gender				
Female	1			
Male	1.06	0.47	2.37	0.89
Mean arterial pressure	0.98	0.94	1.02	0.30
Body mass index	1.03	0.96	1.12	0.42
Triglyceride	1.00	0.999	1.002	0.47
HbA1C	1.02	0.78	1.34	0.90
Diabetic retinopathy				
No diabetic retinopathy (*n* = 46)	1			
Mild NPDR (*n* = 3)	1.01	0.22	4.61	1.00
Moderate NPDR (*n* = 4)	2.76	0.45	16.99	0.27
Severe NPDR (*n* = 2)	1.57	0.20	12.40	0.67
baPWV (cm/s)^*∗*^				
baPWV ≤ 1389.5 (*n* = 10)	1			
1389.5 < baPWV ≤ 1549.5 (*n* = 11)	1.56	0.51	4.79	0.44
1549.5 < baPWV ≤ 1718 (*n* = 16)	3.24	0.95	11.03	0.06
1718 < baPWV (*n* = 18)	4.26	1.08	16.88	0.039

^*∗*^ *p* for trend = 0.025.

**Table 5:** Multivariate logistic regression analysis by quartile baPWV in POAG.

Risk factor	Odds ratio	95% confidence interval		*p* value
Age	0.97	0.89	1.06	0.45
Gender				
Female	1			
Male	0.92	0.19	4.48	0.92
Mean arterial pressure	1.06	0.98	1.14	0.15
Body mass index	0.98	0.78	1.24	0.87
Triglyceride	1.001	0.998	1.003	0.57
HbA1C	0.78	0.39	1.54	0.47
Diabetic retinopathy				
No diabetic retinopathy (*n* = 17)	1			
Mild NPDR (*n* = 3)	3.03	0.43	21.45	0.27
Moderate NPDR (*n* = 0)	—	—	—	—
Severe NPDR (*n* = 0)	—	—	—	—
baPWV (cm/s)^*∗*^				
baPWV ≤ 1378.9 (*n* = 4)	1			
1378.9 < baPWV ≤ 1509 (*n* = 4)	0.69	0.07	6.75	0.75
1509 < baPWV ≤ 1706.3 (*n* = 5)	2.03	0.22	18.55	0.53
1706.3 < baPWV (*n* = 7)	1.99	0.17	22.83	0.58

^*∗*^ *p* for trend = 0.459.
